# Transcriptomic profiling reveals differences in the adaptation of two *Tetragenococcus halophilus* strains to a lupine moromi model medium

**DOI:** 10.1186/s12866-023-02760-w

**Published:** 2023-01-14

**Authors:** Tobias Link, Matthias A. Ehrmann

**Affiliations:** Lehrstuhl für Mikrobiologie, Technische Universität München, 85354 Freising, Germany

**Keywords:** *Tetragenococcus halophilus*, Lupine MRS, Galactose metabolism, Starter culture, Transcriptomics

## Abstract

**Background:**

*Tetragenococcus (T.) halophilus* is a common member of the microbial consortia of food fermented under high salt conditions. These comprises salty condiments based on soy or lupine beans, fish sauce, shrimp paste and brined anchovies. Within these fermentations this lactic acid bacterium (LAB) is responsible for the formation of lactic and other short chain acids that contribute to the flavor and lower the pH of the product. In this study, we investigated the transcriptomic profile of the two *T. halophilus* strains TMW 2.2254 and TMW 2.2256 in a lupine moromi model medium supplied with galactose. To get further insights into which genomic trait is important, we used a setup with two strains. That way we can determine if strain dependent pathways contribute to the overall fitness. These strains differ in the ability to utilize L-arginine, L-aspartate, L-arabinose, D-sorbitol, glycerol, D-lactose or D-melibiose. The lupine moromi model medium is an adapted version of the regular MRS medium supplied with lupine peptone instead of casein peptone and meat extract, to simulate the amino acid availabilities in lupine moromi.

**Results:**

The transcriptomic profiles of the *T. halophilus* strains TMW 2.2254 and TMW 2.2256 in a lupine peptone-based model media supplied with galactose, used as simulation media for a lupine seasoning sauce fermentation, were compared to the determine potentially important traits. Both strains, have a great overlap in their response to the culture conditions but some strain specific features such as the utilization of glycerol, sorbitol and arginine contribute to the overall fitness of the strain TMW 2.2256. Interestingly, although both strains have two non-identical copies of the tagatose-6P pathway and the Leloir pathway increased under the same conditions, TMW 2.2256 prefers the degradation via the tagatose-6P pathway while TMW 2.2254 does not. Furthermore, TMW 2.2256 shows an increase in pathways required for balancing out the intracellular NADH/NADH^+^ ratios.

**Conclusions:**

Our study reveals for the first time, that both versions of tagatose-6P pathways encoded in both strains are simultaneously active together with the Leloir pathway and contribute to the degradation of galactose. These findings will help to understand the strain dependent features that might be required for a starter strain in lupine moromi.

**Supplementary Information:**

The online version contains supplementary material available at 10.1186/s12866-023-02760-w.

## Introduction

**Fig. 1:**
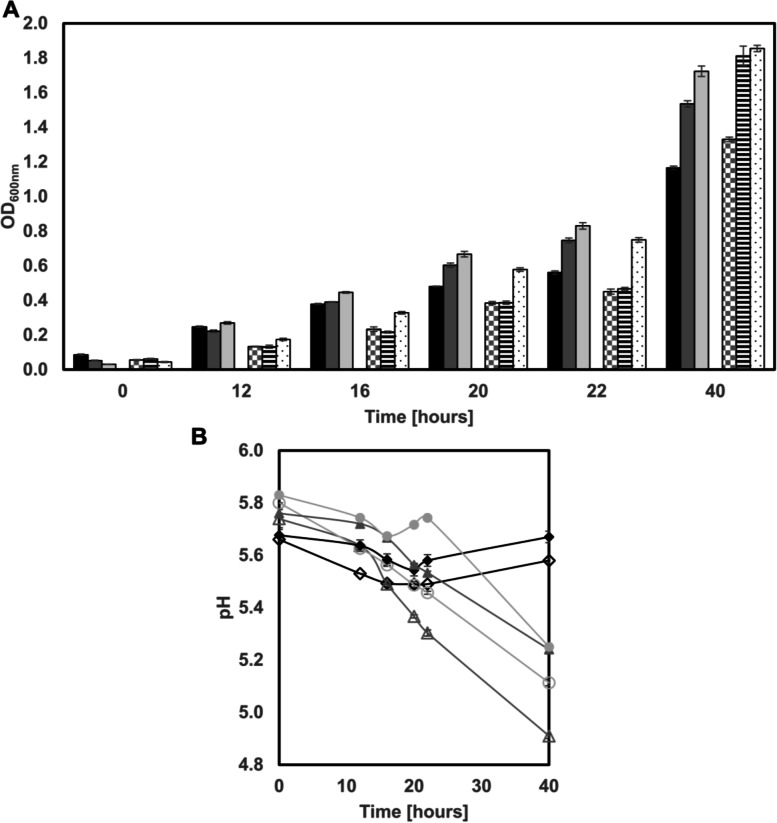
Growth behavior of *T. halophilus* strains TMW 2.2254 and TMW 2.2256 supplied with either D-glucose, D-galactose or no added carbon sources. **A** OD_600nm_ of TMW 2.2254 (black = w/o added carbon sources; dark gray = 10 mM D-glucose; light gray = 10 mM D-galactose); OD_600nm_ of TMW 2.2256 (checked = w/o added carbon sources; black lines = 10 mM D-glucose; black dots = 10 mM D-galactose). **B** pH values of the culture broth of TMW 2.2254 (open symbols) and TMW 2.2256 (filled symbols) (black = w/o added carbon sources; gray = 10 mM D-glucose; light gray = 10 mM D-galactose). Standard deviation is based on 3 biological replicates

*Tetragenococcus* (*T.*) *halophilus* is a moderate halophilic lactic acid bacterium commonly isolated from fermented foods containing high amounts of NaCl, such as lupine seasoning sauce, soy sauce, fermented soybean paste, fish sauce, salted fish but also from different type of cheese [1–6]. *T. halophilus* contributes to the final product by production of organic acids and various volatile compounds [7]. To prevent the growth of spoilers and improve the consistency of the fermentation selected starter strains are added to the respective fermentation [8]. The ability of *T. halophilus* to growth under these conditions is due to the accumulation of compatible solutes (e. g glycine-betaine, choline and proline) and due the ability to increase the intracellular pH by degradation of amino acids such as Arginine and aspartic acid under acidic conditions [9–14]. The species is also known for its diverse carbohydrate utilization patterns, that are in most cases strain specific combinations [15]. This diversity in the carbohydrate utilization pattern might be due to an adaptation to a specific fermentation type or niche within a fermentation [16]. To select starter strains for a new type of fermentation it is important to know what traits are favorable under the given conditions. The production process of the lupine seasoning sauce is similar to the production of traditional soy sauce, but the carbohydrate composition in lupine beans is very different compared to soybeans [17, 18]. Therefore, it is unclear which genetic and biochemical traits are wanted/needed in a starter culture for a lupine seasoning sauce fermentation, especially as lupine beans are rich in galactose oligosaccharides (galactans) [19, 20]. *T. halophilus* has two ways of using this galactose, either via the Leloir pathway or via the tagatose-6P pathway (Fig. 1) [10]. *T. halophilus* is the only species within the genus that possess on a strain dependent basis duplicates or triplicates of the operon encoding for the tagatose-6P pathway, which may indicate an adaptation towards a galactose rich environment in some strains [21]. The fact that multiple gene copies could potentially increase the intracellular gene dosage and thereby increase the fitness on specific substrates was reported for *Cup1* in *Saccharomyces cerevisiae* [22].

In this study, we investigated the transcriptomic profile of two *T. halophilus* strains grown in a lupine peptone-based culture medium supplied with galactose, used as simulation media for a lupine seasoning sauce fermentation. We used two representative strains isolated from lupine moromi that had different growth behaviors in lupine MRS medium (LMRS). The two strains differ in their spectrum of fermentable carbohydrates as TMW 2.2254 is L-arabinose negative, D-melibiose positive and D-lactose negative and TMW 2.2256 is L-arabinose positive, D-melibiose negative and D-lactose positive. Further differences are in the ability to use arginine or aspartate for deamination/decarboxylation, TMW 2.2254 has an inactive ADI-pathway but can utilize aspartate via a specific decarboxylase (*AspD*) and specific transporter (*AspT*). TMW 2.2256 can use L-arginine via the ADI-pathway but does not encode for *AspD* or *AspT* [2]. We therefore wanted to find out if the different growth behavior in LMRS can be explained using transcriptomic analysis. Furthermore, we wanted to find out which of these traits are active and what kind of effects galactose has on the metabolism of each strain.

## Results

### Growth of *T. halophilus* in LMRS

To investigate the growth behavior of *T. halophilus* TMW 2.2254 and TMW 2.2256 in lupine MRS medium (LMRS) we monitored the OD_600nm_ and the pH over 40 h (Fig. 1). Furthermore, to characterize the growth behavior when the major carbohydrate source is altered, growth in LMRS supplied with 10 mM D-glucose or 10 mM D-galactose or no added carbon sources were compared.

Both strains showed a high growth without any extra carbon sources added with an OD_600nm_ of 1.16 or 1.33 for TMW 2.2254 or TMW 2.2256. More surprisingly, the growth was the highest with D-galactose and not with D-glucose for both strains (Fig. 1). TMW 2.2254 reached a final OD_600nm_ of 1.72 (40 h) and TMW 2.2256 reached an end OD_600nm_ of 1.85 after 40 h. With D-glucose TMW 2.2254 reached an end OD_600nm_ of 1.53 and TMW 2.2256 reached an end OD_600nm_ of 1.81 (Fig. 1A).

The pH values with no added carbon sources decreased from 5.66 to 5.49 and then rose back to 5.58 for TMW 2.2254. With the addition of D-glucose the pH decreased from 5.74 to 4.91 over 40 h. Addition of D-galactose led to a decrease from 5.8 to 5.1 of 40 h (Fig. 1B).

The pH values for TMW 2.2256 cultivated in LMRS with no added carbon sources decreased from 5.67 to 5.54 and then rose again to 5.67. The addition of D-glucose led to a decrease from 5.76 to 5.24 over 40 h. D-galactose addition decreased the pH from 5.83 to 5.25 (Fig. 1B).

### Overview of the transcriptomic results

To study the difference of the metabolism of two *T. halophilus* strains when galactose is the main carbohydrate source in LMRS, the strains TMW 2.2254 and TMW 2.2256 were cultivated as biological triplicates in LMRS at 30 °C for 10 h. The growth was monitored by plating serial dilutions of the cultures on to LMRS-5% (w/v) NaCl plates after 1 h and 10 h. The cell count after 1 h was 1.85 × 10^8^ CFU/ml and 1.50 × 10^8^ CFU/ml for TMW 2.2254 and TMW 2.2256, respectively. After 10 h of cultivation the cell count CFU/ml was 4.27 × 10^8^ CFU/ml for TMW 2.2254 and 3.47 × 10^8^ CFU/ml for TMW 2.2256.

For the RNA-seq analysis libraries representing each sample transcriptome were constructed for each strain at timepoints 1 h and 10 h. After sequencing the raw reads were mapped to each genome and normalized after mapping. The uniquely mapped reads for all samples from *T. halophilus* TMW 2.2254 ranged from 22.06 million to 43.54 million and ranged from 24.65 million to 48.13 million for TMW 2.2256. These mapped reads covered 98.3 to 99.4% and 98.7 to 99.7% of the reference genome of TMW 2.2254 and TMW 2.2256. Features with a log2 (fold change) of ≥2 or ≤ − 2 and *p*-value < 0.05 and a false discovery rate (FDR) < 0.01 were considered as DEGs. 121 and 108 differentially expressed genes (DEGs) DEGs were identified for TMW 2.2254 and TMW 2.2256, respectively.

Annotation of DEGs with significant differences revealed that they are involved in biological processes and molecular pathways belonging to carbohydrate and amino acid degradation.

### Differences in the metabolism of carbohydrates and sugar alcohols can mostly be traced back to the galactose utilization

The tagatose-6-P pathway of TMW 2.2254 was increased by 2 to 4.2-fold after 10 h of cultivation. The same pathway was increased by 3.6 to 6.2-fold in TMW 2.2256. Both strains encode for a second version of the pathway with an additional HAD phosphatase, this pathway is here called tagatose-6-P version2. In TMW 2.2254 all enzymes and adjacent transporters were increased by 2.5 to 3.5-fold. In TMW 2.2256 this operon was increased by 2.3 to 5.5-fold. The Leloir pathway was increased in TMW 2.2254 3.4 to 3.7-fold and in TMW 2.2256 2.3 to 3.1-fold. An operon putatively responsible for the utilization of tagatose or fructose via a Tag-1P/Fruc-1P pathway was increased by 4.5 to 5.9-fold in TMW 2.2254 and 5.6 to 6.1-fold in TMW 2.2256. The operon *fruAKR* responsible for the utilization of fructose was 4 to 4.2 and 4.8 to 5-fold decreased in TMW 2.2254 and TWM 2.2256. A mannose-6-phosphate isomerase was increased by 3.1-fold in TMW 2.2254.

A phosphatase part of an operon putatively involved in sucrose metabolism was increased by 2-fold in TMW 2.2256. The Cellobiose operon was only increased (2.9 to 3.5-fold) in TMW 2.2256. The alpha-galactosidase was 2.11-fold increased in TMW 2.2254. The operon *lacEGF* responsible for the lactose metabolism was increased by 6.3 to 8.1-fold in TMW 2.2256. The trehalose operon was increased by 2 to 3-fold and 2.3 to 3.5-fold in TMW 2.2254 and TMW 2.2256, respectively. In both strains a 6-phospho-beta-glucosidase with adjacent PTS-IIA was decreased, 4.2 and 4.3-fold in TMW 2.2254 and 3.9 and 4.2-fold in TMW 2.2256.

The mannitol transporter PTS-IICBA was decreased 3.6-fold in TMW 2.2256. The operon responsible for the glycerol metabolism via the glyercol-3-P pathway (*glpKOF*) was increased 2.2 to.2.7 fold in TMW 2.2256. Another pathway for the metabolism of glycerol is the dehydrogenation pathway via the *gldA*-*dhaMKL* operon. The *dhaMKL* subunits were increased by 2 to 2.3-fold in TMW 2.2256. In TMW 2.2254 the *gldA* was 2.3-fold decreased. Two CDs from the operons putatively responsible for the metabolism of sorbitol was increased by 2 to 2.2-fold.

TMW 2.2254 has two putative glucosamie-6-synthases increased with also increased adjacent PTS systems. The first one consisting of two SIS domain-containing proteins increased by 2.3 and 2.4-fold. The adjacent PTS-IIABCD system was increased by 2.5–2.9-fold. The second operon consisted of a sigma 54-interacting transcriptional regulator (3-fold increase), a PTS-IIABCD (2.9 to 3.9-fold increase), an SIS domain containing protein (2.9-fold increase) and two adjacent chromate transporter (3.4 and 2.8-fold increase). A third operon with similar organization but different domains consisted of a SIS domain-containing protein and adjacent PTS-IIABCD system. The SIS domain-containing protein was increased by 5.4-fold and the PTS system by 4.9 to 5.2-fold.

### Central carbon metabolism

The operon of the citrate lyase *citCDEFX* and an adjacent 2-hydroxycarboxylase transporter were increased in both strains, 2.06 to 2.2-fold in TMW 2.2254 and 2.4 to 3.7-fold in TMW 2.2256. The genomically adjacent oxaloacetate decarboxylase *oadBDGA* was increased in TMW 2.2256 by 2.6 to 3.3-fold, while in TMW 2.2254 only the *oadG* subunit was increased by 2-fold.

Multiple CDS associated with pyruvate metabolism were increased in TMW 2.2256, such as the pyruvate dehydrogenase *pdhABC* (2.3 to 2.6-fold), the pyruvate phosphate dikinase *ppdk* (2.5-fold), the lactate dehydrogenase *ldh* (2.4-fold). Furthermore, a pyruvate:ferredoxin oxidoreductase was increased by 2.2-fold in TMW 2.2256.

A lipoate-protein ligase and adjacent transporter substrate binding domain containing protein were increased by 4.2 and 3.7-fold in TMW 2.2256. An aldose 1-epimerase family protein was decreased by 2.1-fold and a SUF system *NifU* family Fe-S cluster assembly protein was increased by 2.1-fold.

### Differences in the amino acid metabolism in response to LMRS

The arginine deiminase pathway (ADI) of TMW 2.2256 was increased by 4 to 5.8-fold. The 3-phosphoshikimate 1-carboxyvinyltransferase (*aroA*) was decreased by 2-fold in TMW 2.2256. An alanine dehydrogenase *alaD* was increased by 2.1-fold in TMW 2.2256.

An ABC amino acid transporter permease subunit was increased by 2.1-fold in TMW 2.2256. While another ABC amino acid permease subunit and ATP-binding protein was decreased 3.1 to 3.4-fold and 2.9 to 3.2-fold in TMW 2.2256.

### Changes in the nucleotide metabolism and enzymes associated with osmotolerance are strain specific

The cluster responsible for the pyrimidine synthesis consisting of *pyrR* regulator, the uracil permease *uraA*, the small carbamoyltransferase subunit *carA* and the adjacent large carbamoyltransferase subunit *carB* were decreased by 2.1 to 2.6-fold in TMW 2.2256. The anaerobic ribonucleotide-triphosphate reductase activating protein *nrdG* is increased by 2.2-fold in TMW 2.2256. While in TMW 2.2254 the only CDS associated with nucleotide metabolism is the DNA-directed RNA polymerase beta, which is decreased by 2.2-fold.

Osmostress proteins like the betaine-aldehyde dehydrogenase (*gbsA*) and the choline dehydrogenase (*gbsB*) were decreased by 3.9 and 4.3-fold in TMW 2.2254. The iron transporter *fetB* and a zinc ABC transporter substrate binding unit were decreased by 2 and 2.1-fold in TMW 2.2254.

## Discussion

In this study we characterized the growth behavior in a new lupine MRS medium (LMRS) and compared the transcriptomic profile of the two *T. halophilus* strains TMW 2.2254 and TMW 2.2256 to this medium supplied with galactose as the major carbon source. These results should further characterize these strains and reveal strain specific differences that might be beneficial for the growth within the lupine moromi fermentation.

To determine the influence of the major carbon source added to the medium, the growth of TMW 2.2254 and TMW 2.2256 was analyzed when D-galactose, D-glucose or no additional carbon source was added (Fig. 1A, B). Interestingly, both strains grew best with D-galactose. Furthermore, it was also noted that the nutrient concentration in the medium was sufficient to support the growth without the addition of a major carbon source (Fig. 1A). However, it was also notable that the pH values during the cultivation were similar for both strains when no additional carbon source were added, with a small drop at 22 h and then an increase almost back to the start value. The pH values of the cultivations when glucose was added dropped the steepest for both strains. When galactose was added to the medium, the development of the pH values was comparable to the cultivation with D-glucose in TMW 2.2254 although the drop was not as steep and the final pH was higher (Fig. 1B). TMW 2.2256 cultivated with D-galactose the pH values increased after 22 H which could be due to the functional ADI pathway that could help alkalize the cultivation medium. Supporting this hypothesis is the fact that TMW 2.2256 does have a higher pH after 40 h under all conditions compared to TMW 2.2254 (Fig. 1B).

**Fig. 2:**
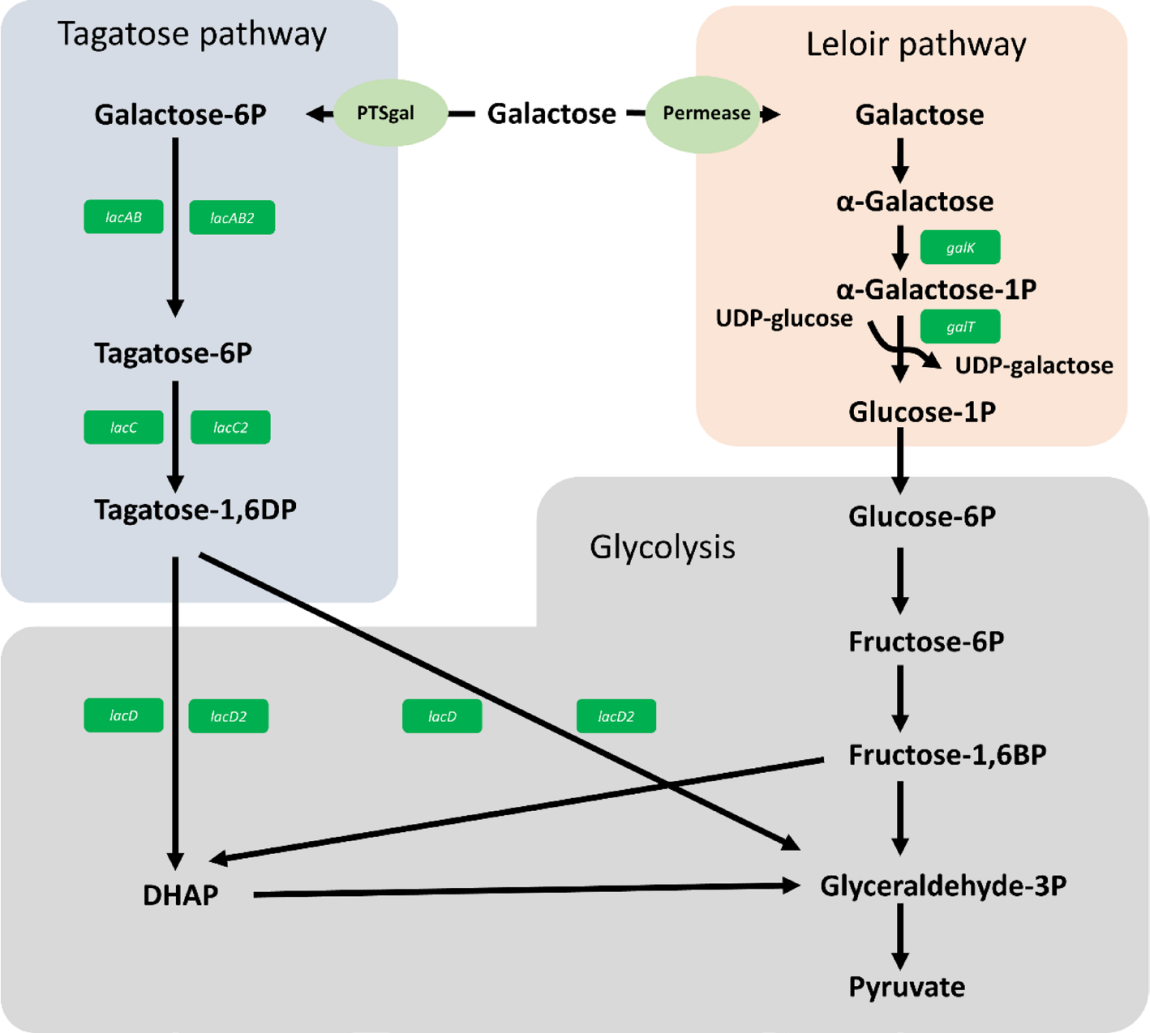
Schematic overview of the significantly upregulated genes of the galactose utilization pathways of *T. halophilus* when cultivated in LMRS supplied D-galactose. Enyzme abbreviations: *galK* = galactokinase; *galT* = UDP-hexose-1-phosphate uridyltransferase; *lacAB/lacAB2* = galactose-6-phosphate isomerase subunit A or B; *lacC/lacC2* = tagatose 6-phosphate kinase; *lacD/lacD2* = tagatose 1,6-diphosphate aldolase; Permease = unidentified permease; PTSgal = Galactose specific PTS systems. Based on Table 1. A KEGG based map including the upregulated genes showing the connection of the genes and substrates can be found in Fig.S1

**Fig. 3:**
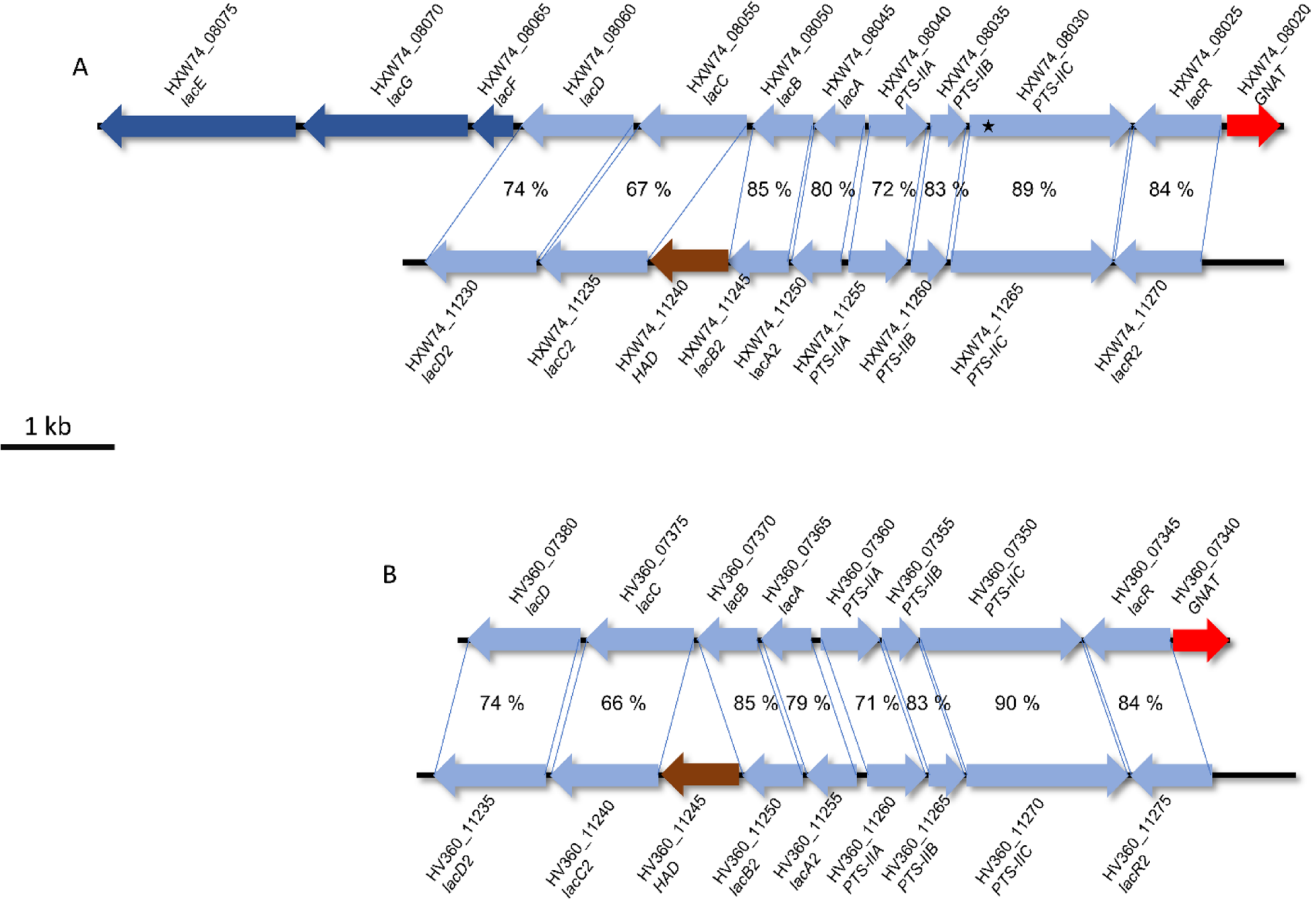
Genetic organization of the CDSs for the tagatose-6P-Pathways in *T. halophilus* TMW 2.2254 and TMW 2.2256. **A** Organization of the tagatose-6P-Pathway *lacCDBAR* and *lacCDBAR2* in TMW 2.2256 with the amino acid identity shown in percent over the entire sequence of the respective CDS; * = indicates a frameshift with a resulting premature stop codon. **B** Organization of the tagatose-6P-Pathway *lacCDBAR* and *lacCDBAR2* in TMW 2.2256 with the amino acid identity shown in percent over the entire sequence. *lacAB/lacAB2* = galactose-6-phosphate isomerase subunit A or B; *lacC/lacC2* = tagatose 6-phosphate kinase; *lacD/lacD2* = tagatose 1,6-diphosphate aldolase*; lacG = 6-phospho-β-galactosidase LacEF* = lactose specific PTS system*; PTS-IIABC*/*PTS-IIABC2* = Galactose specific PTS systems; *lacR*/*lacR2* = putative regulator; *HAD* = HAD family phosphatase; GNAT = GNAT acetyltransferase. The scalebar represent 1 Kb. The sequence similarities are displayed in precent. Alignment was performed using the ncbi blastP program on the ncbi website (https://blast.ncbi.nlm.nih.gov/Blast.cgi?PROGRAM=blastp&PAGE_TYPE=BlastSearch)

As the cultivation media was supplied with 2% D-galactose as main carbohydrate-source, it was expected that this leads to an induction of the pathways responsible for the degradation of D-galactose. The Leloir pathway (*galKETR*) and the tagatose-6-p pathway (*lacDCBAR*) were highly increased in both strains, but a second version of the tagatose-6-P with an additional phosphatase (*lacDCBAR2*) was also highly increased in both strains (Table 1) (Fig. 2). As the CDSs from this second version (*lacDCBAR2*) have a 67 to 90% sequence identity to the first version (*lacDCBAR*), it can be hypothesized that this cluster may have resulted from a gene duplication event (Fig. 3). As this duplicated operon *lacDCBAR2* is active, it could increase the gene doses of the transporters and galactose degrading enzymes and thereby generating a higher overall galactose intake into the cell and explain the higher growth when D-galactose is supplied (Fig. 1A). Therefore, it can be assumed that *T. halophilus* as species in general, is adapted towards a galactose rich environment where multiple copies of galactose degrading enzymes are beneficial. This hypothesis is further strengthened by the fact that several strains from multiple origins have duplicates of this operon, the strain DSM 20338 even contains three copies of this operon [21]. The effect of the additional HAD phosphatase in *T. halophilus* is yet unknown, but it is known that a phosphatase activity enables the use of GAL6P via the Leloir pathway in *Lactococcus lactis* [23, 24]. Therefore, we hypothesis that a similar effect might be present here, although further evidence is needed to completely prove this point. Even more interesting is that TMW 2.2256 when has a higher increase in the abundance of the CDSs of the *lacDCBAR* and of the *lacDCBAR2*, while the Leloir pathway is not as highly increased compared to the fold-changes in TMW 2.2254 (Table 1). This indicates for a preference to utilize the galactose via tagatose-6-p pathway in TMW 2.2256, as the same effect cannot be seen in TMW 2.2254.

**Table 1:**
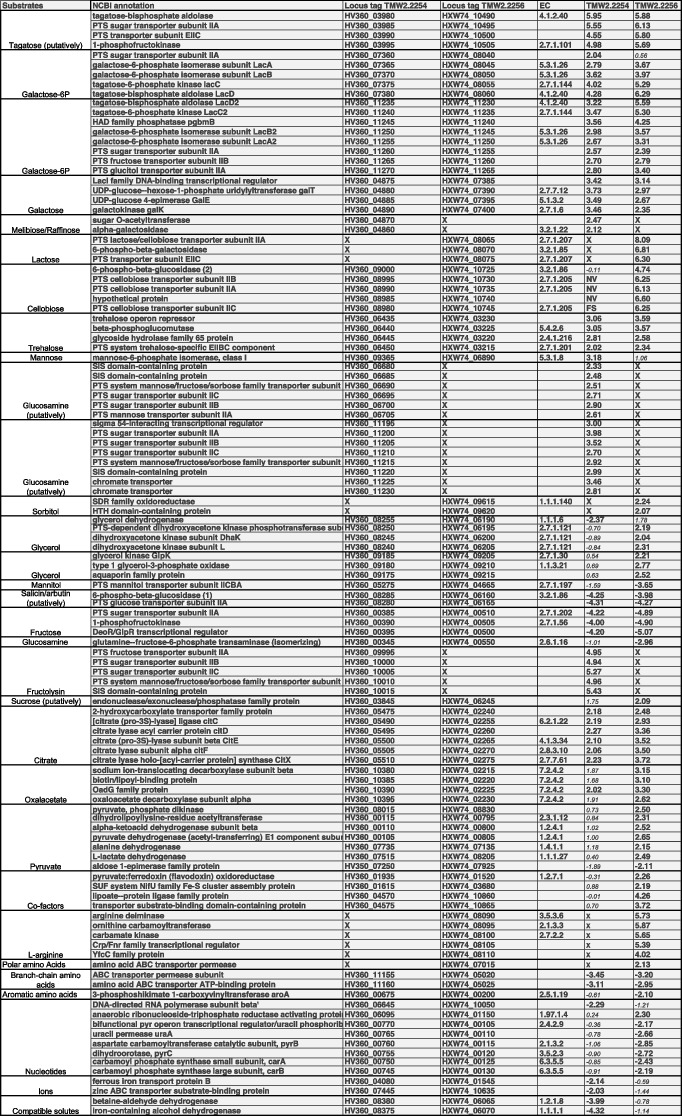
Annotated DEGs from TMW 2.2254 and TMW 2.2256. Annotation of the orfs was done using the NCBI and RAST annotation, but only the NCBI is shown in column two. Substrates and putative substrates of respective genes are listed in column one. The columns three and four contain the respective locus tag in the strain, missing of the ORF is represented by an X. Column four and five show the Log2 fold change of the respective ORF, missing ORFs are represented by X. ORFs that are present in the strain but not count as DEGs are indicated as small italics numbers in white cells. FS indicates and frameshift and a resulting premature stop codon. NV indicates that the comparison of the two conditions yielded no value. Cutoffs: Log2 Foldchange of ≥2 or ≤ − 2, *p*-value ≤0.05 and FDR ≤ 0.01

The strain TMW 2.2254 has three strains specific clusters (HV360_06680-HV360_06705), (HV360_09995-HV360_10015) and (HV360_11195-HV360_11230) consisting of PTS-IIABCD and at least one SIS domain containing protein (Table 1). All of these cluster are at least 2-fold increased. Using the conserved domain search from NCBI, we could assign putative substrates, glucosamine or fructolysine to the genes, but their function in *T. halophilus* grown in LMRS is not clear. However, only two operons are transcribed in a polycistronic manner. The genes HV360_06680 - HV360_06705 are not transcribed polycistronic and therefore can not be considered as an operon.

The operon responsible for the metabolism of cellobiose highly increased in TMW 2.2256 in contrast to TMW 2.2254 (Table 1). A potential explanation could be the fact that the PTS-IIC subunit (HV360_08980) has a premature stop codon and thereby might be not fully functional, rendering this operon inactive. This cellobiose operon might be induced due the residual cellobiose that might be present in the peptone, as the manufacturer stated that 17% residual carbohydrates are present (A230100, Solabia group, France). As the α-galactosidase (α-gal) and β-galactosidase (*lacG*) as well as the adjacent CDSs are strain dependent but increased in the respective strains, the expression might be induced by intracellular galactose levels (Table 1).

The operon responsible for the metabolism trehalose was increased in both strains to a similar degree. However, as trehalose is not added to the medium, we hypothesis that this monocistronic operon is induced by salt stress and trehalose is produced by the trehalose-6-phosphorylase *trePP*, as this reversible reaction to the formation of trehalose can be catalyzed by this enzyme [25]. This is in line with the current knowledge that *T. halophilus* accumulates trehalose intracellularly as a compatible solute under salt stress [26]. Another pathway known for its importance under salt stress in *T. halophilus* is the ADI pathway [10]. This pathway is encoded by the *arcABCRD* operon in TMW 2.2256 but is inactive in TMW 2.2254 as several genes as missing. This pathway generates ATP and NH_3_ by breaking down arginine a thereby increases the intracellular pH. This operon is highly increased with fold-changes up to 5.8-fold as it is induced by salt stress and one of the main repressors, glucose is absent [11].

Strain specific responses can also be found in the pyrimidine synthesis operon *pyrRPBC*-*carAB* (Table 1), as TMW 2.2256 has the complete operon decreased by at least 2-fold and no such changes can be seen in TMW 2.2254. As the bottleneck enzyme *pyrB* in TMW 2.2254 is rendered inactive due to a premature stop codon, the strain completely relies on the uptake of uracil via transporters such as the uracil permease *uraA* and subsequent formation of UMP via the pyrimidine-nucleoside phosphorylase *pdp* and the uridine kinase *udk*. As these enzymes are not regulated by *pyrR*, no changes can be found. However, the decrease of this operon in TMW 2.2256 is most likely due to the regulation of *pyrR*. In *L. lactis pyrR* senses intracellular UMP levels by binding UMP and repressing the correct transcription of the operon under high UMP levels, a similar regulation could be affecting the transcription in *T. halophilus* [27].

The metabolism of sugar alcohols in TMW 2.2254 is negligible as only the glycerol dehydrogenase *gldA* is decreased (Table 1). In contrast to that is the response of TMW 2.2256, in which the entire dihydroxyacetone kinase *dhaMKL* of the dehydrogenation pathway for glycerol as well as glycerol-3-phosphate *glpKOF* pathway is at least 2-fold increased (Table 1). Furthermore, TMW 2.2256 has a SDR family oxidoreductase responsible for the metabolism of sorbitol via a sorbitol-6P 2-dehydrogenase (EC:1.1.1.140) with adjacent PTS-IIA increased for at least 2-fold.

As citrate was added to the LMRS medium in the form of di-ammonium hydrogen citrate (CAS no. 3012-65-5), both strains have the CDSs encoding for the citrate lyase *citCDEFX* increased. Notably, the fold changes in TMW 2.2256 are slightly higher with 2.4 to 3.7-fold compared to TMW 2.2254 with only 2.0 to 2.2-fold changes. As the reaction catalyzed by citrate lyase produces oxaloacetate and acetate, the genomically adjacent oxaloacetate decarboxylase *oadBDGA*, which catalyzes the reaction of oxaloacetate to pyruvate and extrudes sodium ions out of the cell was also increased in both strains [28, 29]. However, in TMW 2.2256 the increase was also higher compared to TMW 2.2254, as in TMW 2.2254 only the gamma subunit was increased by at least 2-fold. As the levels of the citrate lyase are also higher in TMW 2.2256, these differences in the abundance of the CDSs of the *oadBDGA* were expected, as theses enzymes are linked on a metabolic level.

The responses of the strains greatly differ in the pyruvate metabolism, as TMW 2.2256 has the pyruvate-phosphate dikinase *ppdk*, subunit ABC of the pyruvate dehydrogenase, the alanine dehydrogenase *alaD* and a l-lactate dehydrogenase increased by at least 2-fold, while TMW 2.2254 does not. As TMW 2.2256 has more CDSs increased that are linked with the production of pyruvate than TMW 2.2254, the pathways of utilizing pyruvate must then be also increased to a similar degree to keep up with the increased flux of pyruvate. A Portion of pyruvate is most certainly converted to PEP using the ppdk to ensure a high level of PEP in the cell that can be used for the *dhaKML* or for PTS systems. Another portion of pyruvate is converted to acetyl-CoA by the pyruvate dehydrogenase and pyruvate:ferredoxin oxidoreductase. This acetyl-CoA can then be used for formation of acetate or used for the biosynthesis of lipids. The alanine dehydrogenase converts alanine to pyruvate, NH3 and NADH+ H+. This further increases the pyruvate flux and contributes to shifting the balance between NAD+ and NADH+ H+ state inside the cell to side of NADH+ H+. Therefore, an increase of the l-lactate dehydrogenase is needed to regenerate NAD+ to rebalance the redox status (NADH/NAD+ ratio), as the level of the NADH oxidase (HXW74_6500) is constant in TMW 2.2256, the activity of it may be not sufficient to keep up with the increased NADH flux.

Furthermore, the increased transcription and thereby potentially subsequent production of the pyruvate dehydrogenase and pyruvate:ferredoxin oxidoreductase results in an increased need for Fe-cluster assembly proteins and for lipoic acid, as an increase of the assembly protein and lipoic acid transporter can be seen Table 1.

## Conclusion

For the first time it could be shown that all of the operons, in this case two, encoding for the tag-6P in *T. halophilus* are active, which clearly indicates an adaptation towards an environment rich in galactose such as lupine bean moromi or soy sauce moromi. It could be shown that *T. halophilus* is utilizing galactose via two pathways simultaneously and that a preference for either pathway can be a strain dependent feature. The studied strains revealed a different transcriptomic profile, that can be linked to their genomic differences and partially explain the different growth behaviors. Furthermore, the transcriptomic data suggests that TMW 2.2256 has a higher pyruvate flux due more energy active catabolic pathways but also greater redox stress from the resulting shift in the NADH/NAD+ ratio, compared to TMW 2.2254. One of the reasons could potentially be, that by the utilization of sorbitol and glycerol a lot of NADH is generated. TMW 2.2254 is unable to catalyze these reactions and therefore a similar reaction is not required in the same way. Although this study only reveals the difference in two specific strains it shows that the transcriptomic profile can differ greatly between strains. A similar approach might be feasible for other species to determine important traits for growth/persistence in a specific environment.

## Methods

### Strains and cultivation conditions

The strains used in this study were *T. halophilus* TMW 2.2254 and TMW 2.2256. All strains were cultivated in a modified MRS [30] medium, in which the meat extract (10 g/L) and casein peptone (10 g/L) were substituted by the addition of 20 g/L lupine peptone (Solabia, Pantin, France). Then D-glucose or D-galactose or no additional carbon source were added, this medium was given the name Lupine-MRS (LMRS). To simulate the lupine moromi conditions the NaCl concentration were set to 13.5% (w/v) and a pH of 5.7.

### Growth monitoring in LMRS

The growth was characterized by inoculating 15 mL of LMRS, supplied with 10 mM D-glucose or 10 mM D-galactose or without any added carbon sources, with precultures of the respective strains to a starting OD_600nm_ of ≈ 0.05. The growth was monitored over the course of 40 h at 30 °C. The experiment was done in biological triplicates for TMW 2.2254 and TMW 2.2256.

### Cultivation of the cells for the transcriptome analysis

Precultures were grown by inoculating LMRS with 13.5% NaCl (w/v) with single colonies of either strain and incubated statically in a 50 mL falcon tube (Sarstedt, Nümbrecht, Germany) at 30 °C for 48 h. To obtain main cultures for the experiment, 2% of either precultures were used to inoculated 100 mL of LMRS with 13.5% NaCl (w/v) and grown for 24 h under the same conditions.

On the next day, the OD_600nm_ of the main cultures was measured and the volumes required to inoculate 100 mL of LMRS with 13.5% NaCl with approximately 1 × 10^8 CFU/ml of *T. halophilus* were transferred into a 50 mL falcon tube. Then the cells were harvested by centrifugation at 10.000 x g for 10 min at RT and resuspended in fresh 50 mL LMRS. The resuspended solution was then mixed with 50 ml of LMRS in a 125 ml Erlenmeyer flask sealed with a cotton plug and incubated at 25 °C statically for 10 h. The cultures were sampled after 1 h and after 10 h of incubation. All cultivations were done in biological triplicates.

The growth was determined by plating of serial dilutions of a respective sample on to LMRS agar plates with 13.5% NaCl. Therefore, 100 μl of each dilution was streaked out using sterile glass beads (Carl Roth, Karlsruhe, Germany). The plates were then incubated in an anaerobic jar with AnaeroGen™ (Fisher scientific, Waltham, MA, USA) packs at 30 °C. Plates with 20 to 200 colonies were considered for the determination of the cell count.

The cells of the sampled cultures were harvested by centrifugation at 10.000 x g for 10 min at 4 °C and then mixed with 2 mL of RNA-later solution (Thermo Fisher Scientific, Waltham, MA, USA) and kept on ice for 5 min before flash freezing in liquid nitrogen. Samples were then stored at − 80 °C until RNA-isolation. Samples of the supernatant were immediately transferred to a fresh 50 ml tube and then stored at − 80 °C.

### RNA isolation and purification

To isolate RNA from cells the RNAeasy mini kit (Qiagen, Hilden, Germany) was used following the instructions from the manufacturers with some modifications. Frozen samples in RNA-later solution were thawed and the RNA-later solution was discharged without disruption of the cell pellet. Then, the cells were resuspended in 200 μL TE buffer pH 8.5 supplied with 50 mg/ml lysozyme (24,000 kU/mL, SERVA Electrophoresis GmbH, Heidelberg, Germany) for 25 min at RT. The partially lysed cells were then mixed with 700 μL RLT supplied with 2 M DTT and transferred to a fresh 2 ml Eppendorf tube containing acid-washed glass beads 212–300 μm (Sigma Aldrich, St. Louis, MO, USA) and transferred to a homogenizer (MP Fastprep-24,Fisher scientific, Waltham, MA, USA) with a shaking frequency set to 6.5 m/s for 25 s with 5 s pause afterwards, this process was done three times. Next, the lysate was centrifuged at 10. 000 rpm for 10 min at RT and the supernatant was transferred to a fresh 2 ml tube and mixed with 500 μl 96%(v/v) ethanol. After that, 700 μL of the mixture was transferred onto a RNAeasy spin column and proceeded following the manufacturer’s instructions. Quantitative measurement of the RNA content was determined with a Nanodrop spectrophotometer (Nanodrop 1000 3.6.0, PeQLab Biotechnologie GmbH, Erlangen, Germany).

### RNA integrity, sequencing and bioinformatic analyses

RNA integrity analyses, library preparation and sequencing were done by Eurofins genomics. The sequencing was done using an Illumina Hiseq 2500 machine.

Raw read counts were created using featurecounts [31] counting only overlapping “Gene” features with a unique mapping position and minimum mapping quality score of 10. Paired-end reads were only counted if the mapped to the same contig and were counted only once. In the case of reads with multiple mapping results, the reads were assigned to the feature with the highest number of matching bases. The mapped reads were normalized using the Trimmed Mean of M-values (TMM) using edgeR package (version 3.16.5) [32].

The genomes of TMW 2.2254 (GCF_024137165.1) and TMW 2.2256 (GCF_024137145.1) were used as reference for the mapping of the reads using the program BWA-MEM (version 0.7.12-r1039) [33]. StringTie (v. 2.2.1) [34], the annotation using the NCBI PGAP and the proximity of the genes were used for the grouping of genes into operons or clusters. StringTie was used with all settings set to standard.

## Supplementary Information

**Additional file 1.**

## Data Availability

The raw reads data from the transcriptomic experiment are accessible within the Bioproject number PRJNA872913. The genomes of TMW 2.2254 and TMW 2.2256 are accessible under the accession number GCF_024137165.1 and GCF_024137145.1.
