# Supplementary material for: Transcriptomic profiling reveals differences in the adaptation of two Tetragenococcus halophilus strains to a lupine moromi model medium
Source: BMC Microbiol. 2023 Jan 14;23:14. doi: 10.1186/s12866-023-02760-w (PMC9840258; doi:10.1186/s12866-023-02760-w)
Supplement: Supplementary file 1 — Additional file 1. [file 12866_2023_2760_MOESM1_ESM.docx]

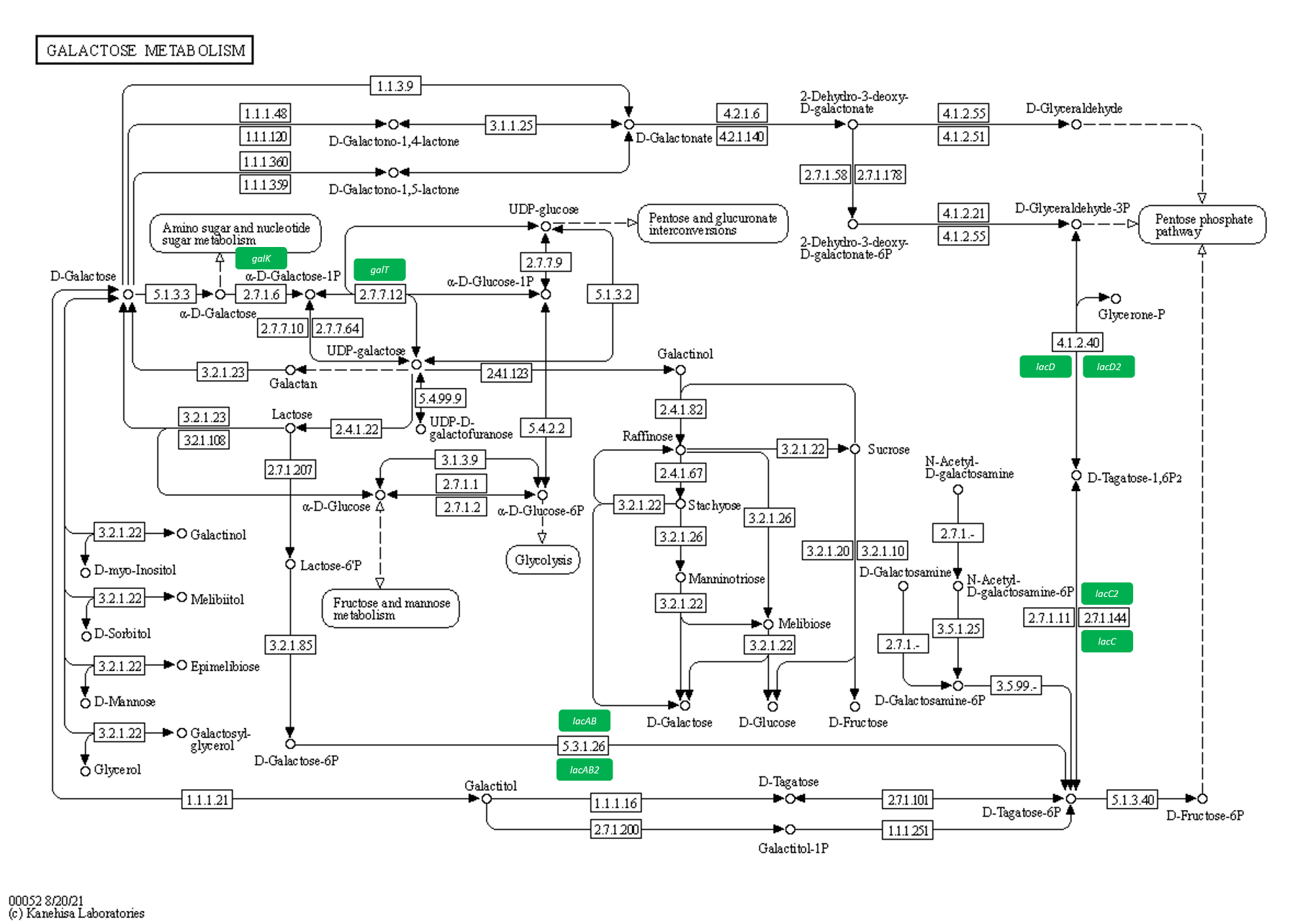


**Fig. S1** Modified map of the galactose metabolism, based on the galactose metabolism reference pathway from KEGG. The significantly upregulated genes of the galactose utilization pathways of T. halophilus when cultivated in LMRS supplied D-galactose are highlighted by a green box above the respective EC numbers. Enyzme abbreviations: galK= galactokinase; galT= UDP-hexose-1-phosphate uridyltransferase; lacAB/lacAB2= galactose-6-phosphate isomerase subunit A or B; lacC/lacC2= tagatose 6-phosphate kinase; lacD/lacD2= tagatose 1,6-diphosphate aldolase. Based on Tab.1.
